# Blue Light Therapy Glasses in Parkinson's Disease: Patients' Experience

**DOI:** 10.1155/2019/1906271

**Published:** 2019-06-18

**Authors:** Katarzyna Smilowska, Daniel J. van Wamelen, Antonius M. C. Schoutens, Marjan J. Meinders, Bastiaan R. Bloem

**Affiliations:** ^1^Radboud University Medical Center, Donders Institute for Brain, Cognition and Behaviour, Department of Neurology, Nijmegen, Netherlands; ^2^Institute of Psychiatry, Psychology & Neuroscience, Department of Basic & Clinical Neuroscience, Division of Neuroscience, King's College London, London, UK; ^3^National Parkinson Foundation International Centre of Excellence, Kings College Hospital NHS Foundation Trust, London, UK; ^4^Chrono Eyewear BV, Tilburg, Netherlands; ^5^Radboud University Medical Center, Radboud Institute for Health Sciences, Scientific Center for Quality of Healthcare, Nijmegen, Netherlands

## Abstract

**Background:**

Blue light glasses have been introduced as a possible new treatment option to treat sleep disturbances in patients with Parkinson disease (PD). Assessing patient attitudes represents a key step in the road towards formal testing and introduction into clinical practice. Specifically, we aimed to assess how patients experience the use of blue light glasses, aiming to optimise compliance in upcoming clinical trials where these glasses will be tested for efficacy.

**Methods:**

We invited 58 PD patients who had used the blue light glasses for at least one week on a daily basis to complete an online survey about their experiences and self-reported impact. For this purpose, the System Usability Scale was used, supplemented with additional questions about (side)effects. A total of 31 patients (53%) replied.

**Results:**

74% of respondents reported subjective improvements in night-time sleep, daytime sleepiness, depressive symptoms, motor functioning, or a combination thereof. The median score for the System Usability Scale (SUS; 0–100 range, higher scores indicating better performance) was 70.0. A total of 26 patients (84%) had an overall positive attitude towards the technique, with patients rating the glasses with an average score of 6.9 ± 2.0 (SD) out of 10. Except for one patient, all responders would like to continue using the glasses, mostly because they considered it a useful aid.

**Conclusion:**

Blue light therapy appears to have a positive effect on sleep, mood, and motor symptoms in PD. PD patients had an overall positive attitude towards blue light glasses as treatment for sleep disorders.

## 1. Introduction

Parkinson's disease (PD) is a neurodegenerative disorder characterized by the coexistence of motor and nonmotor symptoms [1]. Sleep disturbances are among the most common nonmotor symptoms, occurring in up to 90% of PD patients; these sleep disorders reduce quality of life and hamper daytime functioning [2, 3]. Increasing evidence suggests that one of the causes of sleep disorders is perturbation of the suprachiasmatic nuclei, which function as the circadian pacemaker and are responsible for the endogenous physiologic cycles occurring on approximately a 24-hour cycle [4, 5]. Light represents the most effective cue for the circadian timing system, and supplementary exposure to light has beneficial effects on sleep quality and daytime vigilance in healthy older people and patients with dementia [6, 7]. In PD, the circadian system appears to have an impact on motor and nonmotor symptoms. For example, deterioration of motor symptoms (rigidity, tremor, and bradykinesia) reveals abnormal circadian patterns with worsening throughout the day [8, 9]. Distinct diurnal oscillations are also observed in nonmotor symptoms including autonomic dysfunctions, psychiatric symptoms, and sleep impairment. The mechanisms underlying the circadian dysfunction in PD are being increasingly unrevealed, and all evidence clearly points towards the pathological involvement of the suprachiasmatic nuclei [6, 10].

Although many different pharmacological and nonpharmacological strategies have been introduced to manage sleep problems in PD, their efficiency remains limited [11]. Sleep medications often result in side effects and sometimes even in worsening of PD symptoms. Therefore, there is a great need to develop and evaluate nonpharmacological approaches to manage not only sleep disorders in patients but also other symptoms which are related to the perturbation of circadian disruption. One such treatment is bright light therapy (BLT) which is traditionally delivered with light therapy boxes. It is considered as a safe treatment option; only minor side effects have been reported such as headache, eye, or vision-related complains and nausea [12]. However, light boxes are inconvenient and force the patient to stay at one place for at least 30 minutes.

A promising new development is light therapy delivered through light-emitting glasses, instead of traditional light boxes. Additionally, traditional monochromatic white light is being replaced with blue light with a wavelength ranging from 460 to 480 nm. This is more effective in entraining the biological clock [4] and has the potential to phase shift the circadian output better than exposure to white light where longer duration and higher irradiance is needed [5]. The glasses combine both these features as they consist of glasses emitting blue light through integrated LED light sources with a wavelength of approximately 468 nm and a light intensity of 40 Lux at 1.5 cm from the eyes. As a first step to testing the merits of this new approach, we here assessed effectiveness and patients' view on and attitude towards blue light therapy glasses in real life.

## 2. Methods

### 2.1. Survey Design

This online survey was conducted on February and March 2018. All patients who had purchased the blue light glasses were contacted. They were asked to complete an online survey, as outlined below (Appendix 1). The survey included 39 questions, assessing participants' experiences and attitudes toward blue light glasses. The survey included 10 items from the System Usability Scale (SUS) [13, 14], supplemented with 22 questions about the effect of the glasses, including side effects, and baseline characteristics of the participants. Survey responses were all anonymous. The study was approved by the Radboudumc ethics committee.

### 2.2. Study Participants and Data Collection

A total number of 58 patients who were using the blue light glasses on a regular basis were invited to participate in the study. The patients bought the glasses directly form the company producing them (Propeaq; Chrono Eyewear BV, Tiburg, the Netherlands) and agreed to receive research surveys in the future. Other than having a diagnosis of PD and having used blue light glasses for at least one week (as self-reported), there were no formal inclusion or exclusion criteria for participating in this survey.

### 2.3. Statistics

Descriptive statistics were used to summarize all survey data collected in this study. All data are presented as mean ± standard deviation or as percentage where appropriate. All data used to support the findings of this study are available from the corresponding author upon request.

## 3. Results

### 3.1. Patients' Characteristics

A total of 31 out of the 58 patients (53%) completed the survey (20 men; 65%). Average disease duration from PD diagnosis was 6.7 ± 4.6 years. Most patients (28/31, 90%) used PD medication, mainly levodopa preparations. Seven respondents (24%) used sleep medication, consisting of lorazepam (*n* = 1), alprazolam (*n* = 2), clonazepam (*n* = 2), zopiclone (*n* = 1), or melatonin (*n* = 1).

### 3.2. Use of the Glasses

Most patients (23/31, 74%) used the glasses twice daily: once in the morning and once in the evening, for 30–60 minutes each time. All other patients used the glasses once daily in the morning expect for one patient who used the glasses in the evening. On average, patients had been using the glasses for 14.7 ± 12.1 weeks.

### 3.3. Effects of the Glasses

Of the 31 patients, 23 patients (74%) reported an effect of the glasses. Of these, 16 patients (70%) reported an improvement in night-time sleep, four (13%) an improvement in daytime sleepiness, five (16%) an improvement in depressive symptoms, and four (12%) an improvement in motor functioning. Within the group of participants reporting improvements, 18 (78%) used the glasses twice daily, four (17%) once in the morning, and one (4%) once in the evening. A similar distribution was observed in those reported no effect with 50% using the glasses twice daily: 25% in the morning and 25% in the evening.

Five patients (16%) reported adverse events; one patient reported transient cramps in her calves, and two other patients reported transient nausea, transient tiredness, headache, or dizziness.

### 3.4. System Usability Scale

**Figure 1:**
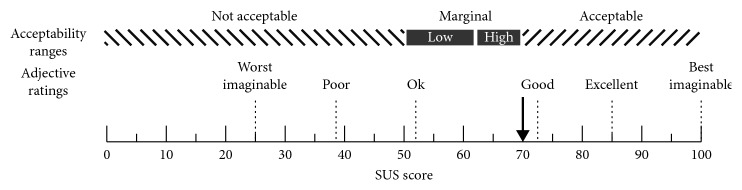
System Usability Scale (SUS) scoring of blue light therapy glasses as rated by participants (*n* = 31), indicated by the black arrow. The median score of 70.0 indicates a good acceptability and classes the glasses between “OK” and “good,” based on the SUS classification.

All participants completed the System Usability Scale. The median score was 70.0 (1^st^ and 3^rd^ QT 57.5–80.0) which classifies the glasses between “ok” and “good” (Figure 1). For patients who noticed an effect of the glasses, the SUS score was 72.1 ± 14.7 (classifying them as “good”); for the nonresponders, the SUS score was 64.1 ± 9.1 (classifying them as “ok”).

### 3.5. Usability of the Glasses

When asked, 25 patients (81%) replied that they either agreed or strongly agreed that they would use the glasses frequently in the future. The other patients had a neutral opinion, except for one patient who disagreed. 21 patients (68%) did not find the glasses unnecessarily complex, and 25 patients (81%) found the glasses easy to use. 19 (61%) patients agreed that the glasses were not cumbersome to use, 26 (83%) patients learned to use the glasses themselves quickly, and 68% (21 patients) also felt confident in using them. Only 45% (14 patients), however, indicated that the glasses were comfortable to wear, whereas only three patients (10%) found using the glasses a stressful event.

### 3.6. Attitude towards the Glasses and the Technique Used

A total of 26 patients (74%) had an overall positive attitude towards the technique. Overall, patients rated the glasses with an average grade of 6.9 ± 2.0, and except for four patients, all patients would continue to use the glasses, mostly because they considered it a useful aid. For patients who noticed an effect of the glasses, the grade given was 7.5 ± 2.5; for the nonresponders, this was 5.4 ± 2.2. 26 patients (84%) would recommend the glasses to other patients.

## 4. Discussion

This was a study on feasibility and patient experiences and not an effectiveness study. Most participants reported that the blue light glasses were acceptable and the attitude towards the technique used in the device was overall positive. Participants reported only minor side effects, and nearly all patients would continue to use the glasses for personal benefit and the sense of contributing to science. In a merely exploratory approach (to inform future larger studies), we did look at some outcomes, and the results showed that 75% of participants reported improvement of PD symptoms, such as sleep and motor symptoms, with some noticing the change as early as within three weeks of use. These findings should be interpreted with great caution, and merely to serve further support, our hypothesized that blue light therapy delivered via glasses might potentially offer a new approach to overcome nonmotor symptoms in PD. To actually demonstrate this, further adequately powered trials with blue light therapy remain needed.

The treatment of sleep problems in PD remains challenging due to side effects of pharmacological treatments, namely, sleepiness, confusion, increased rate of falls, dizziness, and cognitive impairment [15–17]. Only few studies evaluated nonpharmacological interventions, although light therapy has been developed in PD with good results. Blue light is even more effective in influencing the biological clock [4, 18] and in phase shifting of the circadian rhythm [5], and as such represents a promising next step in light therapy.

At first, effectiveness of BLT was confirmed in the treatment of depression in seasonal affective disorder [19]. Then, BLT was offered as nonpharmacological also in neurodegenerative disorder [7, 20]. In PD, BLT aims to improve mood and sleep impairment by shifting the timing of the circadian clock and thereby improving circadian rhythmicity. Light represents the most effective stimulus which is attributed to its Zeitgeber function, which resets circadian rhythmicity reflected in a shift in serum melatonin concentrations [21, 22]. To date, six studies where published with positive results on the effectiveness of BLT on nonmotor symptoms of PD [23–27]. Light therapy was well tolerated in all those studies although none of them tested light exposure via glasses, as we did here. Likewise, in our study, the side effects were minor and included headache, sleepiness, and eye strain [23, 24]. The efficacy of blue light therapy in PD should, however, be confirmed in randomised controlled trials.

The main limitation of this study is the small number of patients responding to the survey, and the fact that the respondents were all users, who bought the classes themselves. Both factors may have caused a bias towards users being more positive about the glasses. Nonetheless, several participants in our survey reported no effect of the glasses and had an overall negative opinion of the glasses, reducing the likelihood of bias in the results we presented here. Moreover, the patients studied here were precisely the ones who would be eligible to use the glasses in either future clinical trials or in daily life. Further studies would benefit from collecting in-depth descriptive statistics and diverse unbiased samples to further understand user opinions and to contribute to the further development of the light glasses.

In conclusion, clinical validation of new digital technologies, with assessment of efficacy, safety, and cost-effectiveness, will be an important part of future research efforts. But understanding the attitudes of patients is particularly useful before such validation can occur and especially prior to any widespread potential clinical implementation. Our results show that PD patients are generally supportive of new technologies, such as the blue light glasses.

## Data Availability

The data used to support the findings of this study are available from the corresponding author upon request.

## Supplementary Materials

Supplementary Materials Appendix 1: questions in the survey.
