# Supplementary material for: Blue Light Therapy Glasses in Parkinson's Disease: Patients' Experience
Source: Parkinsons Dis. 2019 Jun 18;2019:1906271. doi: 10.1155/2019/1906271 (PMC6604290; doi:10.1155/2019/1906271)
Supplement: Supplementary Materials — Appendix 1: questions in the survey. [file 1906271.f1.docx]

**Appendix 1.** Questions in the survey

| **Question** | **Answer or multiple-choice answers** |
| --- | --- |
| **Baseline data** | |
| For how long have you been using the glasses? | In weeks |
| When do you use the glasses | 1. In the morning 2. In the evening 3. Both in the morning and evening |
| For how long at a time do you use the glasses? | 1. Less than 30 minutes 2. Between 30 and 60 minutes 3. More than 60 minutes |
| Do you use medication for your Parkinson disease? | 1. Yes 2. No |
| If you answered ‘yes’ to the above question, which medication do you take? | Open answer |
| Do you use any sleep medication | 1. Yes 2. No |
| If you answered ‘yes’ to the above question, which medication do you take? | Open answer |
| **System Usability Scale** | |
| I think I would use the glasses frequently | 1. Strongly agree 2. Agree 3. Neutral 4. Disagree 5. Strongly disagree |
| I found the glasses unnecessarily complex | 1. Strongly agree 2. Agree 3. Neutral 4. Disagree 5. Strongly disagree |
| I found the glasses easy to use | 1. Strongly agree 2. Agree 3. Neutral 4. Disagree 5. Strongly disagree |
| I feel I need the help of a technical person before I could use the glasses | 1. Strongly agree 2. Agree 3. Neutral 4. Disagree 5. Strongly disagree |
| I found the various functions in the glasses well integrated | 1. Strongly agree 2. Agree 3. Neutral 4. Disagree 5. Strongly disagree |
| I found the use of glasses inconsistent | 1. Strongly agree 2. Agree 3. Neutral 4. Disagree 5. Strongly disagree |
| I imagine that most people would learn to use the glasses very quickly | 1. Strongly agree 2. Agree 3. Neutral 4. Disagree 5. Strongly disagree |
| I found the glasses very cumbersome to use | 1. Strongly agree 2. Agree 3. Neutral 4. Disagree 5. Strongly disagree |
| I felt confident using the glasses | 1. Strongly agree 2. Agree 3. Neutral 4. Disagree 5. Strongly disagree |
| I needed to learn a lot before I could use the glasses | 1. Strongly agree 2. Agree 3. Neutral 4. Disagree 5. Strongly disagree |
| The glasses are comfortable to wear | 1. Strongly agree 2. Agree 3. Neutral 4. Disagree 5. Strongly disagree |
| Using the glasses is stressful to me | 1. Strongly agree 2. Agree 3. Neutral 4. Disagree 5. Strongly disagree |
| **Outcome measures** | |
| Have you noticed any effect by using the glasses? | 1. Yes 2. No |
| If you answered ‘yes’ to the above question, what did you notice? Multiple options possible | 1. Improvement of night-time sleep 2. Improvement of daytime sleepiness 3. Improvement of depressive symptoms 4. Improving of easiness in moving around |
| Have you experienced any side effects? | 1. Yes 2. No |
| If you answered ‘yes’ to the above question, what did you notice? | 1. Headache 2. Nausea 3. Visual problems 4. Other, please specify |
| I have a positive attitude towards the technology used in the glasses | 1. Strongly agree 2. Agree 3. Neutral 4. Disagree 5. Strongly disagree |
| I thought, by using the glasses I could make a valuable contribution to science | 1. Strongly agree 2. Agree 3. Neutral 4. Disagree 5. Strongly disagree |
| Using the glasses gave me more insight into my Parkinson disease | 1. Strongly agree 2. Agree 3. Neutral 4. Disagree 5. Strongly disagree |
| Would you wear the glasses outdoors? | 1. Yes 2. No |
| What grade out of 10 would you score the glasses? 10 is the highest score. | 1.  2.  3.  4.  5.  6.  7.  8.  9.  10. |
| Would you recommend the use of the glasses to other people with Parkinson disease? | 1. Yes 2. No |
| Would you continue to use the glasses? | 1. Yes 2. No |
| If you answered ‘yes’ to the above question, could you indicate why? | 1. I want to continue to contribute to science 2. The glasses are a useful aid for myself 3. I want to continue to discuss the data with my caregiver(s) 4. I want to continue to contribute to the development of the glasses 5. Other, please specify |
| If you answered ‘no’ to the above question, could you indicate why? | 1. Too many technical issues 2. I do not think the technology is useful 3. Personal circumstances 4. Other, please specify |
